# Elastography as a non-invasive method of screening non-alcoholic fatty liver disease in the adult phenotype of paediatric obstructive sleep apnoea

**DOI:** 10.1007/s11325-024-03149-3

**Published:** 2024-09-12

**Authors:** Anna Durdikova, Peter Durdik, Marek Prso, Dominika Dvorska, Lukas Remen, Jarmila Vojtkova, Filip Oleksak, Peter Banovcin

**Affiliations:** 1 Paediatric Department, Jessenius Faculty of Medicine in Martin, Comenius University in Bratislava, Kollarova 2, Martin, 036 01 Slovakia; 2 Paediatric Department, University Hospital Martin, Martin, Slovakia

**Keywords:** Adult phenotype of paediatric obstructive sleep apnoea, Non-alcoholic fatty liver disease, Real-time elastography, Hepatorenal index, Liver fibrosis index

## Abstract

**Purpose:**

The high prevalence of non-alcoholic fatty liver disease (NAFLD) in obese children with obstructive sleep apnoea (OSA) calls for early non-invasive screening. The aim of this study was to use ultrasonographic liver echogenicity and elasticity to evaluate the early stages of liver injury in obese children with OSA.

**Methods:**

Fifty-five obese children with OSA aged 12 to 15 years were included. The control group (*n* = 56) consisted of healthy, non-obese children. All children underwent ultrasound examination to assess liver echogenicity using the hepatorenal index (HRI) and real-time elastography to determine the liver fibrosis index (LFI). Polysomnographic parameters, sonographic values, and clinical-biochemical assessment were statistically analysed according to OSA and its severity. Subgroup 1 was obese children with OSA and AHI < 5 and subgroup 2 was obese children with OSA and AHI ≥ 5.

**Results:**

Higher average values of HRI and LFI were recorded in the group of obese paediatric patients with OSA (mean age ± SD, 14.1 ± 2.2 year; 53% male; BMI z-score, 2.6 ± 0.35) compared to the control group (1.37 ± 0.19 vs. 1.12 ± 0.07, *p* < 0.001 and 1.82 ± 0.31 vs. 1.02 ± 0.27, *p* < 0.001). A significantly higher LFI was recorded in subgroup 2 compared to subgroup 1 (2.0 ± 0.3 vs. 1.6 ± 0.2, p **<** 0.001) while laboratory parameters and HRI (1.4 ± 0.2 vs. 1.4 ± 0.2, p **=** 0.630) did not change significantly. A strong positive correlation was found between the severity of OSA and the LFI (r **=** 0.454; p **<** 0.01).

**Conclusions:**

These findings suggest that ultrasound elastography is a useful non-invasive screening test for OSA-related steatohepatitis in obese adolescents, but other clinical studies are needed to confirm this result.

## Introduction

Paediatric obstructive sleep apnoea (OSA) is characterised by repeated partial or complete collapse of the upper airway during sleep, causing chronic intermittent hypoxia and fragmentation of sleep. This can lead to disruption of the normal oxygenation, metabolic or cardiovascular disease, and obesity [1, 2]. The growing worldwide epidemic of obesity also affects the paediatric population, with an increase in disease related to obesity such as metabolic syndrome, cardiovascular disease, OSA, type 2 diabetes, insulin resistance, and non-alcoholic fatty liver disease (NAFLD) [3]. The prevalence of OSA in obese children is higher than in non-obese children (6% vs. 78%) [4, 5]. Adult phenotype of paediatric OSA refers to a presentation of OSA in children that resembles the characteristics typically seen in adults with OSA. It is characterised by obesity, a short neck, midface hypoplasia several degrees of adenoid and tonsillar enlargement, and often associated with metabolic syndrome and daytime sleepiness [2, 6].

The incidence of NAFLD has increased in children and adolescents over the past decade with the increased prevalence of obesity in paediatric population [7, 8]. NAFLD is the most common chronic liver disease worldwide [7]. The term NAFLD includes benign hepatic steatosis and non-alcoholic steatohepatitis that may develop into progressive hepatic fibrosis and even cirrhosis [7, 9]. Current diagnostic techniques for NAFLD include blood tests and imaging methods, including ultrasonography (USG), computer tomography (CT), magnetic resonance imaging (MRI), and radionuclide scans. Liver biopsy has traditionally been used for determining the degree of fibrosis, though there are some limitations. Transabdominal ultrasound is the most common screening method for NAFLD in clinical practice. However, it is not possible to diagnose mild hepatic steatosis using this technique. Furthermore, classic USG is also unable to distinguish simple steatosis from non-alcoholic steatohepatitis and hepatic fibrosis [10]. It is, however, possible to quantify liver steatosis using USG special software: the degree of steatosis is appraised using the histogram software functionality of the ultrasound device based on measures of the hepatorenal index (HRI). Real-time elastography (RTE) is an advanced ultrasound technology used to image as well as assess the compressibility of liver tissue. It is expressed by the liver fibrosis index (LFI), which seems to be a simple and useful index for assessment of NAFLD [9, 10].

Recently published data have supported an association between NAFLD and OSA in the paediatric population, mostly diagnosed by liver biopsy or laboratory tests [4, 5, 11]. The relationship between these two conditions extends beyond their simple co-existence as obesity-related comorbidities [4, 11]. Therefore, screening for NAFLD in paediatric patients with obesity related OSA is necessary [5]. To our knowledge, there is currently no study available analyzing liver elastography for NAFLD in the adult phenotype of children with OSA. Due to this, we examined the usefulness of liver elastography as a non-invasive screening method for NAFLD in paediatric OSA patients with obesity.

## Materials and methods

### Study population

This prospective cross-sectional study was performed at the Paediatric Sleep laboratory in Martin, Slovakia, between 01/2021, and 01/2023.

Ninety-eight children aged between 12 and 15 years old with obesity and clinical symptoms of OSA were included in this study. Obesity was defined as BMI over the 99th percentile for their age and sex. The typical clinical symptoms of OSA were snoring, sleep disturbance, and witnessed apnoea during sleep. All children had undergone physical examination focused on typical features of adult phenotype and standard overnight in-laboratory video polysomnography (PSG) in the paediatric sleep centre. OSA was defined by an apnoea/hypopnoea index (AHI) greater than or equal to 1 event per hour. Children with an AHI less than 1 or chromosomal syndromes were excluded. A control group of children of a similar age and sex mix, without obesity or OSA excluded by PSG, was recruited from the community. Patients with acute or chronic cardiorespiratory, neuromuscular, or neurologic diseases; congenital craniofacial abnormalities; chromosomal syndrome; or a history of previous treatment for OSA were excluded from the control group. Subsequently, all children from the study and control groups underwent ultrasound examination to quantify liver damage and liver tissue elasticity using the HRI and the LFI. We also performed selected clinical and biochemical assessments in all participants of the study.

The local ethics committee approved the study protocol and the parents of all the children provided informed consent.

### Overnight PSG and sleep stage scoring

All children were evaluated in our paediatric sleep centre by means of a full-night video PSG following an adaptation night in the hospital. Standard overnight PSG recordings were obtained by means of ALICE 6 LDx (Phillips Respironics, Murrysville, PA, USA).

The variables recorded included an electroencephalogram with at least eight channels, an electro-oculogram, a submental electromyogram, and an electrocardiogram. Sleep stages were scored according to the standard criteria of the American Academy of Sleep Medicine (AASM). Chest and abdominal movements were measured by strain gauges. Oronasal airflow was recorded with a thermocouple and, where the child tolerated a nasal cannula, a nasal pressure monitor. Peripheral oxygen saturation was monitored with a pulse oximeter (Masimo Pulse Oximeter Finger Sensor, USA). Central, obstructive, and mixed apnoea events and respiratory-related arousals (RERA) were counted according to the criteria established by the AASM [12]. AHI was calculated as the average number of apnoeas and hypopnoeas per hour of sleep and the diagnosis of OSA was confirmed where AHI was greater than or equal to 1 per hour of total sleep time. Patients with the adult phenotype of OSA were divided into two subgroups according to severity: those with an AHI < 5 (subgroup 1) and those with an AHI ≥ 5 (subgroup 2). ODI (oxygen desaturation index) was calculated as the average number of desaturations of ≥ 3% per hour of sleep.

All recordings were manually and visually scored by one of the investigators (A.D.) and the sleep parameters derived were tabulated for statistical analysis.

### Ultrasound examination of liver

The degree of steatosis was appraised using the histogram software functionality of the ultrasound device (Hitachi Preirus) based on measured HRI values. Simultaneous image capture of liver tissue and the right kidney was obtained using a convex ultrasound probe. With a uniform setting of basic echogenicity, a histogram of the liver and the cortex of the right kidney were realised at the same depth. The HRI value was obtained by finding the ratio of liver and kidney echogenicity medians. If the HRI was less than 1.2, steatosis was excluded. An HRI between 1.2 and 1.5 was considered to be non-significant steatosis, corresponding to fewer than 5% of hepatocytes being affected on histological examination of a biopsy. Significant steatosis corresponding to more than 5% of hepatocytes is recognised at HRI values ≥ 1.5 with 100% sensitivity and 91% specificity. Steatosis in more than 25% and 60% of hepatocytes is observed at HRI values greater than 1.89 and 2.23, respectively. These cut-off values were standardised in adults with steatosis verified by tissue biopsy [13]. Determination of liver tissue elasticity was carried out using the same ultrasound device with real-time elastography software. A convex ultrasound probe was attached to the 5–7 intercostal space in the mid-axillary line. The LFI was determinate from displayed liver elasticity map and calculated by ultrasound software. Final LFI values were calculated as an average of three measurements [9, 13].

### Statistical analysis

The variables were statistically analysed by SYSTAT11. Data from group was first tested for normality and equal variance by the Shapiro-Wilk test. All data were normally distributed and presented as mean ± standard deviation (SD). Descriptive statistics were used to describe demographic and anthropometric parameters of patients and controls. The chi-squared test and unpaired Student’s t-test were used to establish differences between variables. Differences between the means of selected parameters measured during PSG recording, ultrasound examination, and elastography were assessed using the independent non-paired Student’s t-test. The Pearson correlation coefficient was used to correlate the severity of OSA in case groups to parameters of ultrasound elastography parameters. A p-value of less than 0.05 was considered to be statistically significant. A correlation coefficient (r) between 0 and 0.19 was considered weak; 0.20–0.39 mild; 0.40–0.59 moderate; 0.60–0.79 moderately strong; and 0.80-1.0 as strong correlation.

## Results

### Study groups

From 98 subjects in study group 43 children were excluded from the study: 33 were had an AHI less than 1; 7 children had chromosomal syndromes; and 3 refused ultrasound examination of the liver. Following these exclusions, 55 children subsequently fulfilled the criteria for adult phenotype of paediatric OSA. In this group, the mean age was 14.1 ± 2.2 years; 53% were male; and the mean BMI z-score was 2.56 ± 0.35. 56 healthy controls were of similar age (14.6 ± 2.4 years) and sex mix (53% male). There were statistically significant differences in BMI z-score (2.56 ± 0.35 vs. 0.12 ± 0.36; *p* < 0.001); neck circumference (27.9 ± 2.6 vs. 34.6 ± 3.9; *p* < 0.001); waist circumference (112.2 ± 14.5 vs. 73.9 ± 11.2; *p* < 0.001); and hip circumference (122.5 ± 17.0 vs. 89.5 ± 3.16; *p* < 0.001) between the case and control groups. No statistically significant difference was found in selected laboratory parameters between these two groups. The demographic, anthropometric, and laboratory profile of the study population groups has been summarised in Table 1.

**Table 1:** Demographic, clinical, and biochemical features of the study population, grouped according to the presence/absence of OSA and obesity

Parameters	Unit	Control group	Obese OSA group	*p*-value
Sex: Female Male	n (%)	27 (48%) 29 (52%)	26 (47%) 29 (53%)	*p* = 0.371
Age	years ± SD	14.6 ± 2.4	14.1 ± 2.2	*p* = 0.471
Anthropometric Parameters				
Height	cm ± SD	168.9 ± 14.0	163.6 ± 16.8	*p* = 0.231
Weight	kg ± SD	53.5 ± 22.1	104.5 ± 28.7	*p* < 0.001
BMI	kg/m^2^	20.05 ± 17.1	38.8 ± 7.5	*p* < 0.001
BMI z-score	z-score	0.12 ± 0.36	2.56 ± 0.35	*p* < 0.001
Neck circumference	cm ± SD	27.9 ± 2.6	34.6 ± 3.9	*p* < 0.001
Waist circumference	cm ± SD	73.9 ± 11.2	112.2 ± 14.5	*p* < 0.001
Hip circumference	cm ± SD	89.5 ± 13.6	122.5 ± 17.0	*p* < 0.001
Laboratory tests				
AST	µkat/l	0.5 ± 0.4	0.5 ± 0.5	*p* = 0.592
ALT	µkat/l	0.5 ± 0.5	0.6 ± 1.0	*p* = 0.533
GMT	µkat/l	0.4 ± 0.4	0.5 ± 0.4	*p* = 0.871
ALP	µkat/l	2.8 ± 2.6	3.0 ± 1.4	*p* = 0.912
TAG	mmol/l	1.4 ± 0.6	1.6 ± 0.7	*p* = 0.924
HDL	mmol/l	1.1 ± 0.9	1.1 ± 0.3	*p* = 0.312
LDL	mmol/l	2.6 ± 0.9	2.8 ± 0.8	*p* = 0.242
Cholesterol	mmol/l	4.2 ± 1.2	4.6 ± 1.1	*p* = 0.181
Glucose	mmol/l	4.9 ± 1.2	5.3 ± 0.9	*p* = 0.243
Total bilirubin	µmol/l	9.9 ± 8.1	12.0 ± 8.8	*p* = 0.361
Vitamin D	µg/l	15.5 ± 13.5	14.7 ± 5.6	*p* = 0.572
CRP	mg/l	4.5 ± 2.1	5.2 ± 2.9	*p* = 0.784

*BMI*, body mass index: *AST*, aspartate aminotransferase: *ALT*, alanine aminotransferase: *GMT*, gamma-glutamyltransferase: *ALP*, alkaline phosphatase: *TAG*, triacyglycerol: *HDL*, high-density lipoprotein: *LDL*, low-density lipoprotein: *CRP*, C-reactive protein: *OSA*, obstructive sleep apnoea

We further categorised the cohort of patients with obesity according to the severity of OSA into two subgroups: subgroup 1 (33 children with obesity and OSA with an AHI < 5; age 14.3 ± 2.1 years; BMI z-score 2.37 ± 0.31) and subgroup 2 (22 obese OSA children with AHI ≥ 5; age 14.00 ± 2.5 years; BMI z-score 2.65 ± 0.35). There was a significant difference between BMI (35.0 ± 4.80 vs. 41.36 ± 9.28; *p* < 0.01), and BMI z-score (35.0 ± 4.80 vs. 41.36 ± 9.28; *p* < 0.01) parameters between subgroup 1 and subgroup 2. There was no significant difference of laboratory parameters between these two subgroups of adolescents (Table 2). The differences in selected respiratory parameters of PSG are shown in Table 3.

**Table 2:** The clinical and biochemical features of the study population, grouped according to the severity of OSA

Parameters		Subgroup 1: Obese OSA, AHI < 5	Subgroup 2: Obese OSA, AHI > 5	*p*-value
Number of patients	n (%)	33 (60%)	22 (40%)	
Age	years ± SD	14.3 ± 2.1	14.0 ± 2.5	*p* = 0.716
Anthropometric Parameters				
Height	cm ± SD	169.6 ± 12.9	160.8 ± 21.0	*p* = 0.206
Weight	kg ± SD	101.9 ± 22.7	107.4 ± 37.0	*p* = 0.646
BMI	kg/m^2^	35.0 ± 4.8	41.4 ± 9.3	*p* < 0.05
BMI z-score	z-score	2.37 ± 0.31	2.65 ± 0.35	*p* < 0.05
Neck circumference	cm ± SD	34.1 ± 3.2	35.6 ± 5.1	*p* = 0.371
Waist circumference	cm ± SD	109.1 ± 13.5	113.9 ± 16.1	*p* = 0.431
Hip circumference	cm ± SD	119.7 ± 13.7	123.9 ± 21.6	*p* = 0.553
Laboratory tests				
AST	µkat/l	0.7 ± 0.7	0.5 ± 0.2	*p* = 0.481
ALT	µkat/l	0.9 ± 1.3	0.6 ± 0.4	*p* = 0.427
GMT	µkat/l	0.5 ± 0.3	0.6 ± 0.4	*p* = 0.933
ALP	µkat/l	3.1 ± 1.7	3.0 ± 1.0	*p* = 0.933
TAG	mmol/l	1.6 ± 0.7	1.6 ± 0.7	*p* = 0.925
HDL	mmol/l	1.0 ± 0.2	1.2 ± 0.4	*p* = 0.238
LDL	mmol/l	2.6 ± 0.5	3.0 ± 1.1	*p* = 0.231
Cholesterol	mmol/l	4.4 ± 0.5	5.0 ± 1.5	*p* = 0.192
Glucose	mmol/l	5.5 ± 0.9	5.0 ± 0.8	*p* = 0.237
Total bilirubin	µmol/l	14.3 ± 10.7	10.0 ± 3.9	*p* = 0.245
Vitamin D	µg/l	14.9 ± 5.5	13.5 ± 6.1	*p* = 0.568
Glycated haemoglobin	%	3.6 ± 0.5	3.64 ± 0.5	*p* = 0.778
Insulin	pmol/l	24.3 ± 15.3	26.0 ± 8.9	*p* = 0.760
CRP	mg/l	4.1 ± 2.5	5.9 ± 3.4	*p* = 0.137

*BMI*, body mass index: *AST*, aspartate aminotransferase: *ALT*, alanine aminotransferase: *GMT*, gamma-glutamyltransferase: *AL*, alkaline phosphatase: *TAG*, triacyglycerol: *HDL*, high-density lipoprotein: *LDL*, low-density lipoprotein: *CRP*, C-reactive protein: *OSA*, obstructive sleep apnoea

**Table 3:** Respiratory polysomnographic parameters of study population, grouped according to the severity of OSA

Polysomnographic parameters	Subgroup 1 Obese OSA, AHI < 5	Subgroup 2 Obese OSA, AHI > 5	*p*
AHI	2.0 ± 1.6	26.7 ± 20.6	*p* < 0.0001
ODI	1.6 ± 1.3	28.2 ± 29.9	*p* < 0.002
Average saturation (%)	94.3 ± 1.1	93.0 ± 3.2	*p* = 0.144
Arousal index (AI)	15.9 ± 7.3	56.2 ± 62.1	*p* < 0.02
Respiratory AI	0.7 ± 1.1	31.0 ± 51.6	*p* < 0.03

*OSA*, obstructive sleep apnoea: *AHI*, apnoea hypopnoea index: *ODI*, oxygen desaturation index: *AI*, arousal index

### Ultrasound profile

**Fig. 1:**
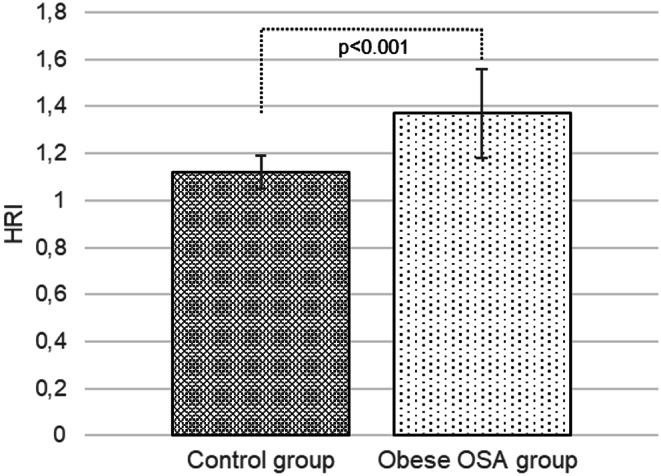
Comparison of hepatorenal index between the control and the obese group with obstructive sleep apnoea, *MSOffice*

**Fig. 2:**
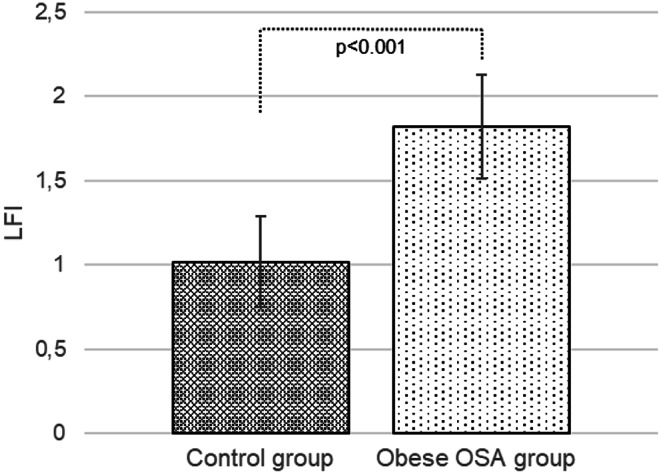
Comparison of liver fibrosis index between the control and the obese group with obstructive sleep apnoea, *MSOffice*

Higher mean HRI and LFI values were recorded in the group of obese paediatric patients with OSA compared to the group of healthy non-obese children without OSA (1.37 ± 0.19 vs. 1.12 ± 0.07, *p* < 0.001 and 1.82 ± 0.31 vs. 1.02 ± 0.27, *p* < 0.001) (See Figs. 1 and 2).

**Fig. 3:**
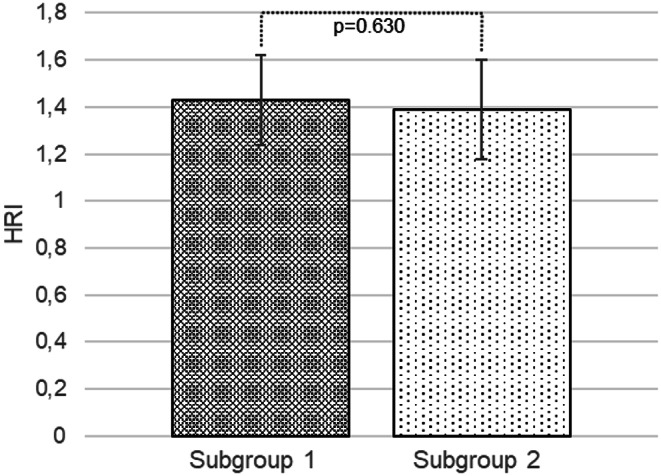
Comparison of hepatorenal index between the obese subgroup with obstructive sleep apnoea and apnoea hypopnoea index < 5 events per hour of total sleep time and the obese subgroup with obstructive sleep apnoea and apnoea hypopnoea index ≥ 5 events per hour of total sleep time, *MSOffice*

**Fig. 4:**
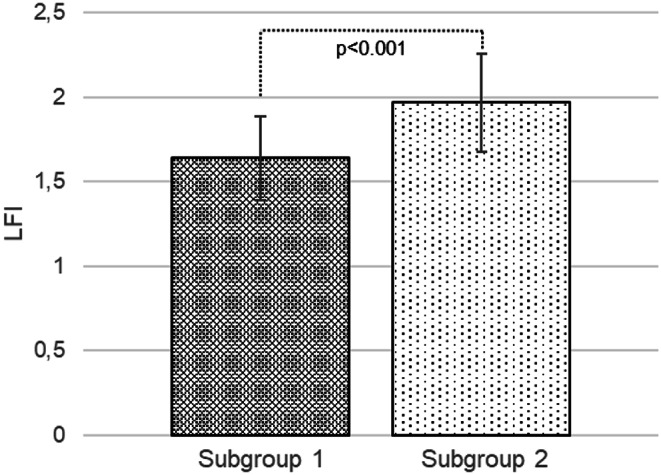
Comparison of liver fibrosis index between the obese subgroup with obstructive sleep apnoea and apnoea hypopnoea index < 5 events per hour of total sleep time and the obese subgroup with obstructive sleep apnoea and apnoea hypopnoea index ≥ 5 events per hour of total sleep time, *MSOffice*

Measurements of sonographic values revealed differences in obese patients with OSA. Significantly higher values of LFI were recorded in subgroup 2 compared to subgroup 1 (1.97 ± 0.29 vs. 1.64 ± 0.25, *p* < 0.001), but there was no significant difference in HRI (1.39 ± 0.21 vs. 1.43 ± 0.19, *p* = 0.630). Subsequently, HRI and LFI values were compared between subgroup 1 and the control group, and statistically significantly higher values ​​of HRI (1.43 ± 0.19 vs. 1.12 ± 0.07, *p* < 0.001) and LFI (1.64 ± 0.25 vs. 1.02 ± 0.27, *p* < 0.001) were recorded in subgroup 1. A strong positive correlation was found between the severity of OSA and LFI (*r* = 0.454; *p* < 0.01). Obese patients with more severe OSA had a higher LFI, especially those with AHI > 5  (See Figs. 3 and 4).

## Discussion

NAFLD and OSA, both serious complications of obesity, have become prevalent in childhood [9]. Recent studies have reported a link not only between and cardiometabolic complications, but also between OSA and both direct (hepatic histology) and indirect - serum alanine aminotransferase (ALT) or gamma-glutamyltransferase (GMT) markers of liver damage in morbidly obese patients with NAFLD in both adult and paediatric populations [11]. Therefore, children with OSA should be screened for NAFLD, and those with NAFLD should be screened for OSA [3].

A wide spectrum of biomarkers has been proposed for use in the diagnosis of NAFLD, as well as for quantifying the degree of fibrosis. However, liver biopsy remains the main diagnostic and staging tool. Early studies which assessed the prevalence of NAFLD in children based on aminotransferase levels or standard ultrasonography estimated a prevalence of between 3 and 7% [14]. Studies performed in obese children showed a higher prevalence of elevated ALT, ranging between 8 and 42%, while the prevalence of a bright liver on standard ultrasonography, indicating liver steatosis, ranged between 1.7 and 77% [15]. However, the measurement of ALT and/or imaging techniques to detect the prevalence of NAFLD seems to not be accurate indicators of fibrosis. Some patients with mildly increased or normal ALT levels have been found to have substantial fibrosis [16]. Standard ultrasound lacks the sensitivity to identify steatosis in the setting of macrovesicular fat, which affects less than 30% of hepatocytes [17].

Elevated serum aminotransferases are present in 20 to 50% of adults with OSA. On histological examination there is more severe hepatic inflammation in obese adults with severe OSA and hypoxaemia than those with OSA without hypoxaemia [18]. A study of a selected sleep medicine clinic population reported that 91% of obese children with elevated aminotransferases have OSA, although liver histology was not evaluated [18, 19]. OSA is also associated with NAFLD, evidenced by elevated liver enzymes and progressive hepatic fibrosis in the paediatric population [5]. Moreover, circulating selected markers of liver apoptosis and inflammatory markers are elevated in children with OSA [20]. Verhulst et al. confirmed a relationship between sleep disordered breathing and presumed NAFLD based on the presence of obesity and elevated AST, ALT, and/or hyperechoic liver ultrasonography. In these studies, elevated aminotransferases were primarily used to determine the presence of NAFLD, although children with NAFLD may have normal AST and ALT results [21]. We did not find a significant difference in aminotransferases between obese patients with OSA and the control group, as well as neither in both subgroups.

Standard ultrasonography as a tool for the detection of liver disease has a sensitivity of 79.7% and a specificity of 86.2%. However, hepatic steatosis may be difficult to visualise using normal ultrasonography in subjects with central adiposity, and 30% hepatic fat may be necessary for detection using this imaging modality [22].

Data on the relationship between NAFLD and OSA in young people with obesity are sparse [11, 23]. Despite this, we screened our OSA patients for the early stages of liver injury using elastography. Elastography-based imaging has shown promising results in this age group as a potential new, non-invasive replacement of the liver biopsy [14, 24]. Most studies performed in the adult population with NAFLD have shown that elastography is useful for ruling out the presence of fibrosis, as well as for differentiating between stages of fibrosis [24]. Marginean et al. showed that liver stiffness values measured from transient elastography (TE) were significantly higher in children with obesity compared to those with normal-weight ones [14]. Also, Cho et al. reported that this method is reliable for screening obese Japanese children for steatohepatitis and liver fibrosis [25]. Studies performed on children with biopsy proven NAFLD have concluded that TE is accurate for the prediction of the presence of liver fibrosis regardless of its severity [26].

In our study we used a screening tool for the early stages of liver injury secondary to NAFLD in our paediatric patients. However, the cut-off values used were validated for adults with steatosis confirmed by tissue biopsy. There is a lack of studies in the paediatric population in this field. We found significantly higher mean HRI and LFI values in the study group of obese paediatric patients with OSA compared to the control group. Measurements of sonographic values also revealed differences between obese patients with OSA of different severities. There was a significantly higher LFI in the subgroup of obese children with more severe OSA (AHI ≥ 5) compared to those with mild OSA (AHI < 5). There was no difference in HRI between the two subgroups. A strong positive correlation was found between the severity of OSA and LFI, but not between OSA severity and HRI. Obese patients with more severe OSA had a higher LFI, particularly those in subgroup 2 with an AHI $\:\ge\: 5$. Although most paediatric patients with obesity and OSA had a more echogenic liver on sonography, children in subgroup 2 were more prone to steatohepatitis (elevated LFI). Determination of liver elasticity seems to be a suitable and sufficiently sensitive tool for detecting suspected liver inflammatory processes in children with OSA and obesity. Sunduram et al. demonstrated that OSA and hypoxaemia are common in paediatric patients with NAFLD confirmed by liver biopsy [18]. Animal model studies have found that chronic intermittent hypoxia, considered to be the main characteristic of OSA, leads to liver damage likely through stimulation of the production of oxygen-derived compounds [11]. In their review, Mann et al. state that oxidative stress is central to the pathogenesis of NAFLD. [27]. Sundaram et al.’s study included 31 adolescents with both OSA and NAFLD confirmed by biopsy. These children may trend toward more severe fibrosis. In children with NAFLD, increased sleep hypoxia and sleep apnoea is associated with higher hepatic steatosis, histological grade of inflammation, and NAFLD Activity Score. The strongest risk factor for the exacerbation of NAFLD and fibrosis aggravation in patients with OSA is the severity of chronic intermittent hypoxia [28]. Microbiota may also be involved in steatohepatitis related to OSA. Chronic intermittent hypoxia may exacerbate gut microbiota dysbiosis and therefore contribute to the exacerbation of NAFLD [29].

The current standard treatment of NAFLD in children and adolescents is lifestyle change, with the aim to decrease caloric intake and increase physical activity [7]. However, screened obese children and adolescents for OSA and NAFLD seem to be crucial for management both of conditions. Adequate treatment of OSA improved both obesity and NAFLD, and vice versa. Untreated OSA patients experience more difficulty losing weight. The evidence currently available suggests that treating OSA with CPAP will not reverse an exacerbation of NAFLD, although it may slow or stabilise disease progression and have metabolic and cardiovascular benefits in adults. The crucial task seems to be the combination of several therapeutic goals, particularly long-term CPAP treatment [30]. On the other hand, a study by Sundaram et al. provided strong evidence that treatment of OSA and nocturnal hypoxia with CPAP in children with NAFLD may reverse parameters of liver injury and reduce oxidative stress. CPAP appears to be a new therapy for the prevention of progression of NAFLD in children with obesity found to also have OSA [18].

Our study has some limitations. Its cross-sectional nature prevents the definition of a causal relationship between OSA and NAFLD in children. A low number of patients were included in the study group. Real-time elastography (RTE) provides a quantitative measure of liver stiffness. This method differs from other US-based elastography techniques in that it does not provide a quantitative estimate of liver stiffness [24]. Further longitudinal studies in children are needed to confirm our results and to monitor improvements of elastographic parameters in patients with OSA undergoing CPAP treatment.

## Conclusion

Ultrasound elastography seems to be a useful and non-invasive diagnostic method to screen for steatohepatitis in the adult phenotype of paediatric OSA. However, further clinical studies are needed to confirm this result. In addition to the early detection of these changes, we can assess the dynamics of the disease and the effect of applied therapy non-invasively and in real-time, what improves the overall control over this pathology. In the future these new non-invasive diagnostic options could minimise the need for liver biopsy, which may help to avoid the possible procedural biopsy associated risks.

## Data Availability

The data that support the findings of this study are not openly available due to reasons of sensitivity and are available from the corresponding author upon reasonable request. Data are in controlled access data storage at Paediatric department of Jessenius Faculty of Medicine.

## Declarations

### Submission declaration and verification

The authors confirm that this manuscript has not been published elsewhere, is not under consideration by any other journal and the paper has been professionally proofread.
